# Evaluation of the immunological functions of placental alkaline phosphatase *in vivo* using ALPP transgenic mice

**DOI:** 10.3389/fimmu.2025.1499388

**Published:** 2025-02-06

**Authors:** Tse-Ching Chen, Kwai-Fong Ng, Ning Chen, Yi-Ling Pan, Chun-An Cheng, Hsiao-Chun Wu, Yi-Jen Hsueh, Nien-Yi Chiang, Hsi-Hsien Lin

**Affiliations:** ^1^ Department of Anatomic Pathology, Chang Gung Memorial Hospital-Linkou, Taoyuan, Taiwan; ^2^ Department of Ophthalmology and Center for Tissue Engineering, Chang Gung Memorial Hospital-Linkou, Taoyuan, Taiwan; ^3^ Kennedy Institute of Rheumatology, University of Oxford, Oxford, United Kingdom; ^4^ Department of Microbiology and Immunology, Graduate School of Biomedical Sciences, College of Medicine, Chang Gung University, Taoyuan, Taiwan; ^5^ Division of Rheumatology, Allergy and Immunology, Chang Gung Memorial Hospital-Keelung, Keelung, Taiwan; ^6^ Center for Molecular and Clinical Immunology, College of Medicine, Chang Gung University, Taoyuan, Taiwan

**Keywords:** placental alkaline phosphatase, phagocytosis, sepsis, skin graft rejection, transgenic mouse

## Abstract

Alkaline phosphatase (ALP) is a ubiquitously expressed dephosphorylating enzyme and its level in blood is widely used as a diagnosis marker of liver damage or bone disorders in human patients. ALP is also considered as an anti-inflammatory protein due to its ability to dephosphorylate and inactivate inflammation-triggering molecules such as lipopolysaccharide (LPS). Placental alkaline phosphatase (ALPP) is one of tissue-specific ALP isozymes expressed mostly during pregnancy, however it was found to be differentially upregulated in certain hepatocellular carcinomas by us recently. In addition, ALPP has been identified as a reliable biomarker of diverse germ cell tumors. Nevertheless, little is known of its immune modulatory role *in vivo*. In this study, we generated ALPP transgenic mice and tested these mice in the LPS-induced sepsis and male-to-female skin graft rejection models. Our results showed that ALPP transgenic mice are more susceptible to intraperitoneal injection of LPS in comparison to control animals. In addition, female ALPP transgenic mice were better at delaying the rejection of male skin grafts. In an *in vitro* phagocytosis experiment, addition of exogenous ALPP compromised the phagocytic ability of THP-1 monocytic cells. These results indicate that excess ALPP plays a role in modulating both innate and adaptive immune functions.

## Introduction

Alkaline phosphatase (ALP) is a dephosphorylating enzyme capable of hydrolyzing the phosphate moiety from various biological compounds such as nucleic acids, phospholipids and phosphoproteins (1–3). ALP is expressed ubiquitously in most prokaryotic and eukaryotic organisms, including *E. coli* and human (3, 4). In human, ALP is derived mostly from liver and bone, and plays an important role in the physiological functions of several essential tissues/organs, such as metabolic regulation within liver and skeletal mineralization for bone turnover (1, 5–8). In clinical settings, ALP is recognized now as a reliable diagnostic and prognostic marker for several human diseases and hence a routine monitor of blood ALP level is often recommended.

On the other hand, ALP is capable of reducing inflammation by dephosphorylating some inflammation-triggering molecules such as bacterial lipopolysaccharides (LPS) and extracellular nucleotides (9–13). These inflammation-triggering molecules are pro-inflammatory signals that may lead to undesirable local and/or systemic inflammation, inducing septic shock, when not controlled stringently (14, 15). Importantly, the parenteral administration of ALP to patients with severe sepsis showed a significant improvement of renal function in several clinical trials, suggesting a potential role for ALP in immune modulation *in vivo* (16–18).

Interestingly, four distinct ALP isozymes encoded by as many different genes are expressed in human. These ALPs are divided into two categories, namely the tissue-nonspecific and tissue-specific types (19, 20). The tissue-nonspecific ALP (also called ALPL) is expressed mainly in liver, bone, and kidney by a gene on the chromosome 1 (21). On the other hand, the tissue-specific ALPs, including those expressed in intestine (intestinal ALP, ALPI), placenta (placental ALP, ALPP) and germinal tissues (germinal ALP, ALPG), are produced by genes located on the chromosome 2 (22). Normally, ALPL represents the predominant ALP isozyme in circulation, but the tissue-specific ALPs may also contribute to the serum ALP pool under specific conditions.

Among the tissue-specific ALP isozymes, ALPP is expressed primarily in the placenta, especially syncytiotrophoblasts and primordial germ cells, starting in early weeks of gestation and continuing to increase its expression throughout pregnancy (23, 24). ALPP is a glycosylated membrane-bound dimeric enzyme tethered to the cell surface via the glycosylphosphatidylinositol (GPI) anchor (25). In addition, ALPP could also be released from cell membrane as a secretory protein following the digestion by specific phospholipases. Moreover, ALPP has also been identified as a biomarker of various germ cell tumors such as seminoma and dysgerminoma (26, 27). These findings altogether suggest a physiological function unique to placenta during pregnancy for ALPP. Additionally, our recent study identified unusually elevated ALPP expression in human hepatocellular carcinomas with enhanced motility, suggesting a pro-tumorigenic role that extends beyond its placenta-specific functions (unpublished results). Given the immune modulatory role of ALP in septic infection, we decided to investigate the systematic effect of ALPP on the immune responses of LPS-induced sepsis and skin transplantation *in vivo* by establishing a transgenic animal model of ALPP over-expression. Our results indicate that ALPP transgenic mice are more susceptible to LPS-induced sepsis and are more resistant to male-to-female skin graft rejection. Moreover, the phagocytic ability of THP-1 monocytic cells was attenuated by the addition of exogenous ALPP. In summary, we conclude that ALPP plays a modulatory role in both innate and adaptive immune systems.

## Materials and methods

### Generation of ALPP transgenic mice

The expression construct containing the full-length human ALPP cDNA in the pCMV6-XL4 vector (TrueClone™, NM_001632, SC119167) was purchased from OriGene Technologies, Inc. (Rockville, MD). To generate transgenic mice constitutively expressing human ALPP, an ALPP expression vector was constructed by inserting the full-length ALPP cDNA into the pCAGGS vector (Addgene, Cambridge, MA) via the *EcoR I* and *Xba* I/*Bag* I restriction sites. DNA sequencing was performed to confirm the authentication of the ALPP cDNA fragment of the final construct. The pCAGGS-ALPP construct was linearized and purified for pronuclear injection of mouse embryos as described elsewhere. Generation of the ALPP-transgenic mice was carried out in the National Laboratory Animal Center, Taiwan using standard procedures as described (28) and verified by genotyping PCR analysis of tissue DNA samples using specific ALPP primers (5’-GAGGACTCGGGATCTTCAGG-3’ and 5’-GGGACTACAGGCGCATAGAT-3’). Briefly, fertilized eggs (zygotes) were collected from superovulated donor FVB/N female mice after mating with stud males. The zygotes were carefully washed in medium and microinjected with purified transgenic DNA (2 ng/μL) using an automated microinjector set to a constant flow rate of 50 hPa. Microinjected zygotes were cultured overnight until they reached the two-cell embryo stage, then transferred into pseudo-pregnant recipient mice. Pregnancies were monitored, and the recipient mice were expected to deliver pups after approximately 20 days. The pups were routinely observed for normal development, and a small tissue biopsy was collected around 10 days after birth for DNA isolation and genotyping to confirm the transgenic founder animals. After verification, the founder animals were bred with wild-type C57/BL6 mice to establish the transgenic mouse line. The mice were housed under a 12-hour light/dark cycle in a controlled environment with a constant temperature of 22°C and 55 ± 10% humidity. They had unrestricted access to a standard rodent diet and water. The animal experiments have been reviewed and approved by the Institutional Animal Care and Use Committee (IACUC) of Chang Gung Memorial Hospital-Linkuo, Taiwan (Approval No. 2022032901). Details of all reagents are provided in **Supplementary Table 1**.

### Animal model of LPS-induced septic shock

Sex-matched control C57BL/6 (B6) and ALPP-transgenic mice of similar ages (8 to 12 wk-old) were injected intraperitoneally (ip) with 15 mg/kg of LPS from *Escherichia coli* serotype O111:B4 (Sigma Chemical Co.), which produced a 30% lethality in pretested animals. Mice were monitored at least twice daily for endotoxemia by assessing signs such as reduced activity, unsteady gait, diminished appetite, and increased eye secretions. The progression of endotoxemia was ultimately determined by animal mortality.

### Skin grafting experiments on mice

Grafting of male mouse skin onto female mice was conducted according to a modified technique of Billingham et al. (29). Briefly, female B6 and ALPP-transgenic mice were anesthetized with a mixture of 10 mg/ml Hypnodil and 2 μg/ml Sublimaze (Janssen). Full thickness tailskin (1 × 1 cm) from male mice was grafted onto the lateral flank of female mice. Grafts were observed on alternate days after the removal of the bandage at day 8 post-transplantation and considered rejected when no viable donor skin was present. Statistical analysis of graft survival was made using the log-rank test with Prism 6 edition (30).

### *In vitro*phagocytosis assay

THP-1 cells were cultured in RPMI medium containing 10% fetal calf serum (FCS) and supplemented with glutamine, β-mercaptoethanol, amino acids, penicillin, and streptomycin. The phagocytosis assay of THP-1 cells was carried out using the pHrodo™ Green BioParticles^®^ Phagocytosis Kit (Invitrogen, Grand Island, NY, USA) exactly as suggested by the manufacturer and measured by the flow cytometry analysis. Briefly, THP-1 cells (5 x 10^5^ cells/well) were incubated without or with 200 nM recombinant human ALPP (rhALPP)(OriGene Technologies, Inc, catalog no: TP310504) for 24 h before adding pHrodo™ Green BioParticles conjugated *E. coli* (20 μL/well) and incubated at 37°C for 24 h. The optimal absorption and fluorescence emission maxima of the pHrodo™ Green BioParticles is approximately 509 nm and 533 nm, respectively. The assay protocol was followed as per manufacturer instructions. The data was acquired by using BD FACS calibour and analyzed by cell quest pro software.

## Results

### ALPP transgenic mice are more susceptible to LPS-induced sepsis

ALPP transgenic mice were generated with no significant difficulty and housed in the same animal facility with the wild-type C57/BL6 mice. The exogenous human ALPP cDNA sequence was routinely verified by PCR analysis to ensure its transmission to offspring through successful breeding of the transgenic mouse lines (**Supplementary Figure 1**). These results indicate that ALPP over-expression did not affect the overall development of the animals. In addition, both gross examination and histopathological analysis of kidney, liver, spleen, adrenal, thymus, heart and GI tract of ALPP-transgenic mice revealed no apparent abnormalities (**Supplementary Figure 2**). Flow cytometric analysis of splenocytes of the ALPP transgenic mice showed normal percentage of T cells, Foxp3^+^ regulatory T cells, B cells, NK cells, macrophages, dendritic cells and CD34^+^ stem cells (**Supplementary Table 2**). In conclusion, the development and basal functions of the *in vivo* immune system in general seems to be normal in the presence of excess ALPP.

**Figure 1:**
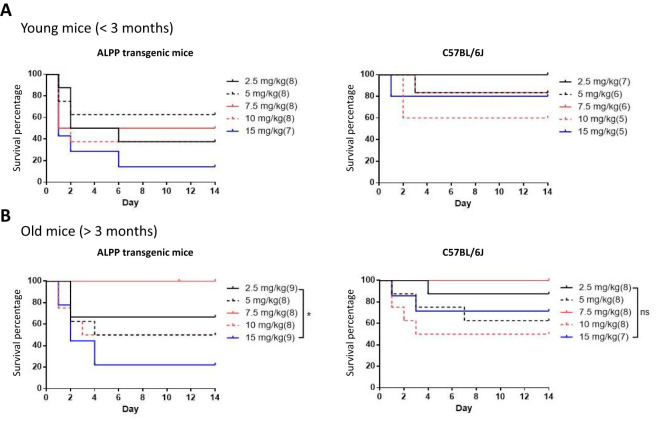
Dose-dependent LPS-induced septic shock death in young **(A)** and old **(B)** WT B6 mice and ALPP transgenic mice. The data showed that older mice in general were more susceptible to LPS-induced sepsis than the younger mice.

**Figure 2:**
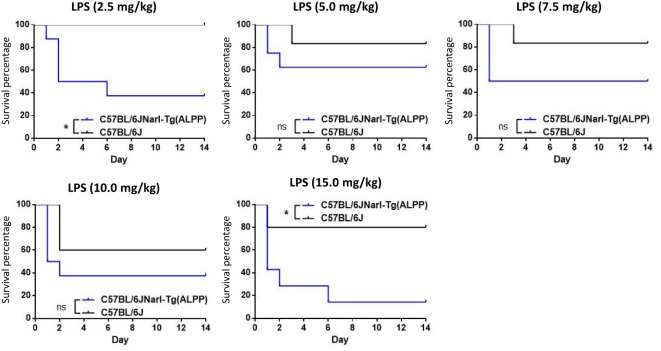
ALPP transgenic mice were more susceptible than C57/BL6 mice to LPS-induced sepsis in a direct comparison of younger animals receiving the ip injection of same LPS concentrations. Data were derived from at least 5 animals per group and presented as means ± SEM. Two-way ANOVA: *p < 0.05. ns: non-significant.

To test the role of ALPP in acute inflammatory responses *in vivo*, we first treated transgenic mice and wild-type (WT) B6 mice with different concentrations of ip injected LPS. As shown in **Figure 1**, both transgenic mice and B6 mice were sensitive to LPS treatment in a dose-dependent manner in younger (< 3 months of age) and older (> 3 months of age) animals. Furthermore, the older mice were found to be more susceptible to LPS than the younger mice, as expected. Interestingly, comparable analyses of the younger mice receiving the same LPS doses showed that ALPP transgenic mice were more vulnerable than the WT B6 mice to LPS-induced sepsis (**Figure 2**). We conclude that high levels of ALPP exacerbate *in vivo* systematic acute inflammatory responses induced by LPS, likely affecting the effector functions of innate immune cells.

### ALPP compromises the *in vitro* phagocytotic ability of monocytes

**Figure 3:**
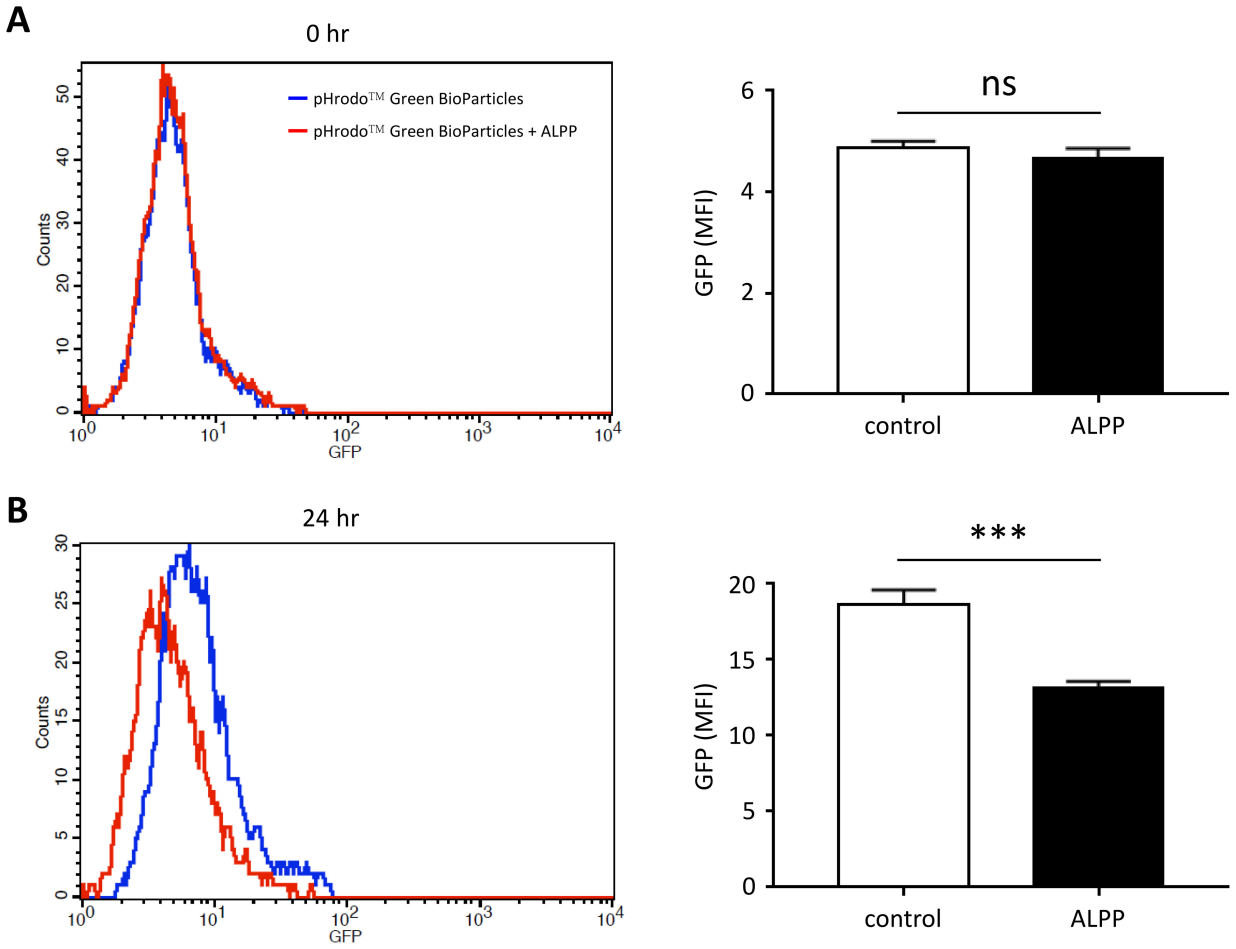
The effect of exogenous ALPP on the phagocytic ability of THP-1 cells. Cells were treated without or with 200 μM recombinant human ALPP before adding the pHrodo™ Green BioParticles^®^ for 0 hr **(A)** and 24 hr **(B)**. The extent of phagocytosis was determined by the green fluorescence within cells by flow cytometry analysis. Data were derived from 3 independent experiments and presented as means ± SEM. ns, non-significant; ***p < 0.001. Count, cell number; GFP, fluorescence intensity; MFI, mean fluorescence intensity.

To evaluate the effect of ALPP on innate immune cell functions, we tested the phagocytic ability of THP-1 cells in the absence or presence of excess recombinant ALPP. As shown in **Figure 3**, human THP-1 monocytic cells expectantly internalized fluorescence-labelled inactivated, unopsonized *E. coli* bioparticles upon a period of incubation. Interestingly however, attenuated phagocytic activities were clearly noted when cells were treated with exogenously added ALPP. These data indicate that ALPP negatively regulates the phagocytic activity of monocytic innate immune cells *in vitro*.

To investigate the immune modulatory effect of ALPP on LPS-induced sepsis and the phagocytic activity of monocytic cells, we carried out a cytokine multiplex assay to measure serum levels of specific pro-inflammatory and anti-inflammatory cytokines in LPS-treated mice. As shown in the **Supplementary Figure 3**, the results revealed that G-CSF levels at 1, 3, and 5 hrs post-LPS administration were similarly elevated in both wild-type C57BL/6 and ALPP transgenic mice. However, at 7 hours, G-CSF levels were significantly higher in the ALPP transgenic mice compared to the wild-type group. Notably, no significant differences were observed in the levels of the other eight cytokines, including GM-CSF, IL-1β, IL-4, IL-6, IL-10, IL-13, IFN-γ, and TNF-α. Taken together, these findings suggest that ALPP likely exerts its immune modulatory effects through distinct yet unknown mechanisms.

### Female ALPP transgenic mice showed increased resistance to male skin graft rejection

**Figure 4:**
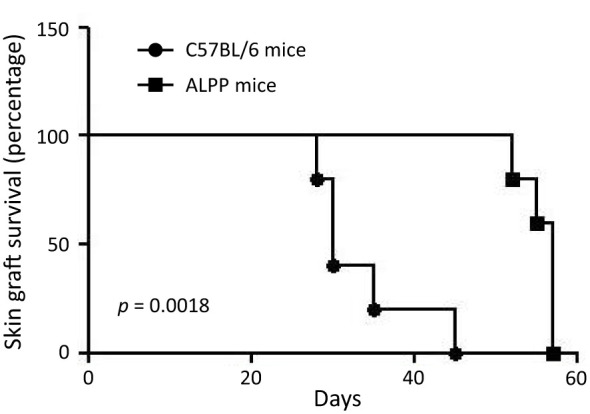
The results of skin graft survival in female wild-type C57/BL6 and ALPP transgenic mice receiving male skin transplantation. 5 C57/BL6 and 5 ALPP transgenic mice were included in the male-to-female skin graft rejection experiment and were monitored for up to 60 days post-transplantation for skin graft survival.

Rejection of tissue/organ allotransplantation results from a series of complex interactions involving various innate and adaptive immune cell types, with T cells playing a central role in this process. Hence, T cell-mediated adaptive immune responses to the allografts represent a major obstacle to a successful tissue transplantation. To investigate the role of ALPP in adaptive immune responses, we next employ an *in vivo* male-to-female skin graft rejection model. In brief, female WT B6 and ALPP transgenic mice were subjected to tissue grafting of male mouse tail skin onto the lateral flank. The occurrence of skin graft rejection was monitored continuously for up to 45 days. Our results showed that while all 5 WT mice rejected male skin grafts by 45 days post-transplantation, the male skin grafts remained stably viable in the 5 female ALPP transgenic mice even until after the 50^th^ day of tissue transplantation (**Figure 4**). Specifically, the male skin grafts on female ALPP transgenic mice were rejected between the 52^nd^ and 58^th^ days after transplantation.

## Discussion

In this study, we attempted to investigate the immunoregulatory functions of ALPP, which is a GPI-anchored glycosylated dimeric ALP isozyme expressed primarily in the placenta (22, 23). As a result of the GPI linkage, ALPP is shed conditionally from cell surface and secreted into circulation. In pregnant women, both membrane-bound and soluble ALPP might have local and systemic functional effects during pregnancy. Interestingly, our preliminary findings showed elevated ALPP expression by human hepatocellular carcinomas, suggesting a possible pregnancy-independent tumorigenic role (unpublished results). To explore this, we first studied the *in vivo* immune function of ALPP by establishing and testing ALPP transgenic mice in an animal model of LPS-induced septic shock. Our results showed that ALPP transgenic mice are in fact more susceptible to LPS-induced sepsis and lethality in comparison to the WT B6 mice (**Figures 1**, **2**). As LPS-induced sepsis is caused by systemic acute inflammatory responses derived mainly from innate immune cell types including macrophages and neutrophils, these results indicate that consistently high levels of ALPP *in vivo* exacerbate the inflammatory reactions induced by LPS-activated innate immune cells. Although previous studies have suggested that ALP may act as an anti-inflammatory dephosphorylating enzyme of some inflammation-triggering molecules such as LPS and extracellular nucleotides, other uncharacterized substrate molecules of ALPP likely exist and may function to enhance the overall inflammatory reactions induced by LPS. Moreover, ALP has been implicated in regulating other physiological functions, including purinergic signaling, metabolic pathways, and the composition of the intestinal microbiome (15, 31, 32). These mechanisms may potentially explain the heightened LPS-induced lethality observed in ALPP transgenic mice.

In an attempt to further dissect the possible role of ALPP in modulating innate immune cellular functions, the phagocytic ability of THP-1 monocytic cell was examined. As expected, THP-1 cells actively internalized *E. coli*-bioparticles in the absence of ALPP. Interestingly however, the phagocytic activities of THP-1 cells were attenuated upon ALPP treatment, suggesting an impaired phagocytic function (**Figure 3**). This result suggests an inhibitory role for ALPP in the bacterial phagocytosis of THP-1 cells, but the mechanism whereby ALPP reduces the phagocytic ability remains to be deciphered.

Finally, we tested the possible effect of ALPP in the adaptive immune system by using an *in vivo* male-to-female skin graft rejection model. Female mice usually reject male skin graft within 45 days, but the skin grants in female ALPP transgenic mice was not rejected even after 55 days post-transplantation (**Figure 4**). As T cells are the key immune cell types responsible for allograft rejection, these results indicate that ALPP could probably also impair T cell functions. In summary, our *in vivo* and *in vitro* experiments indicate that ALPP is involved in regulating both the innate and adaptive immune functions, in part by affecting the cellular functions of macrophages and T cells. While our current results point to a novel biological function of ALPP in immunomodulation, a detailed mechanistic study focusing on ALPP-targeted protein substrates and cellular signaling mechanisms shall provide insights into the molecular pathways driving its role in inflammation and its contribution to disease progression.

## Data Availability

The original contributions presented in the study are included in the article/**Supplementary Material**. Further inquiries can be directed to the corresponding author.
