# Supplementary figures and images for: Evaluation of the immunological functions of placental alkaline phosphatase in vivo using ALPP transgenic mice
Source: Front Immunol. 2025 Feb 6;16:1499388. doi: 10.3389/fimmu.2025.1499388 (PMC11839614; doi:10.3389/fimmu.2025.1499388)

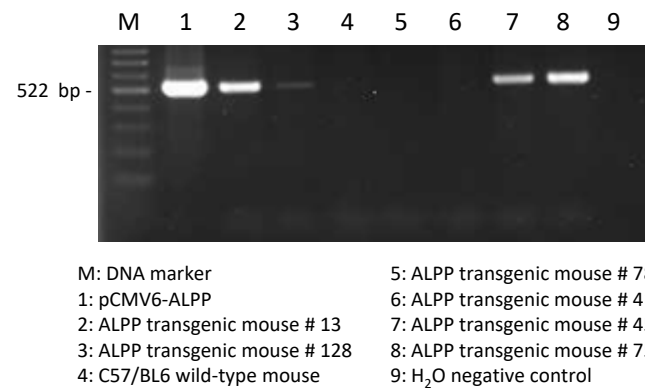

**Supplementary Fig. 1**

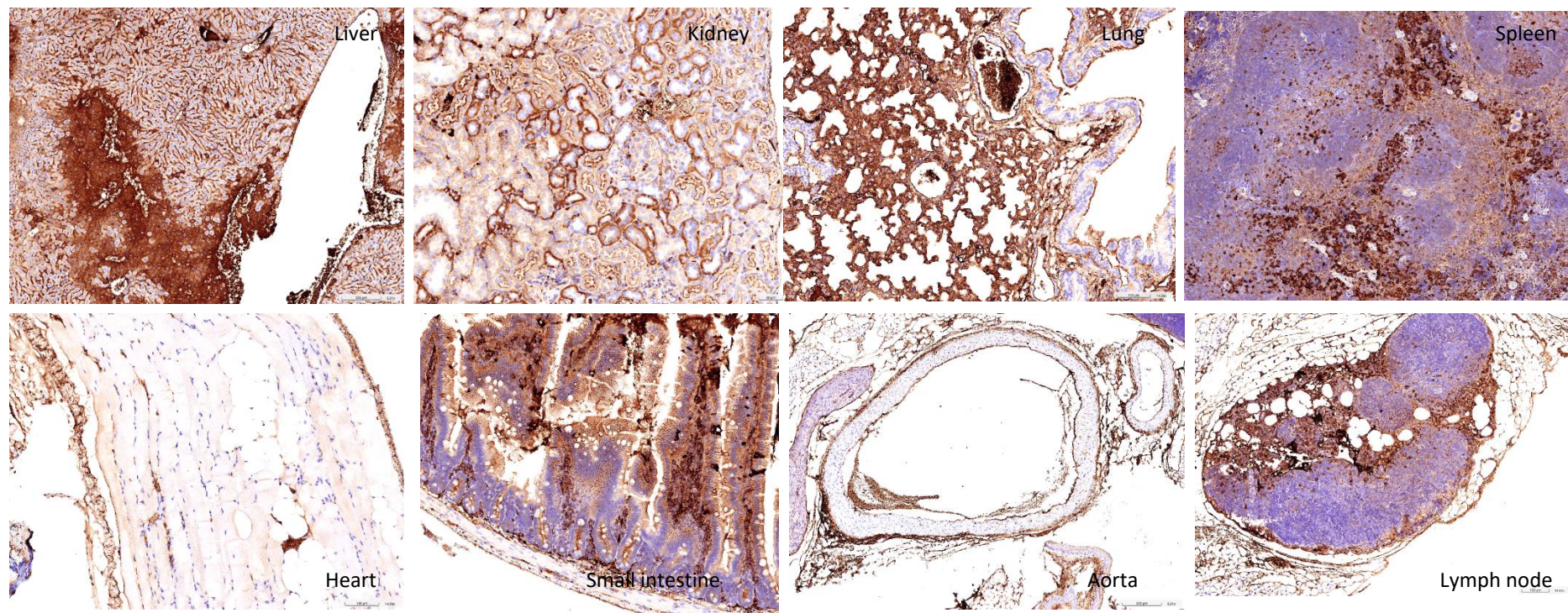

**Supplementary Fig. 2**

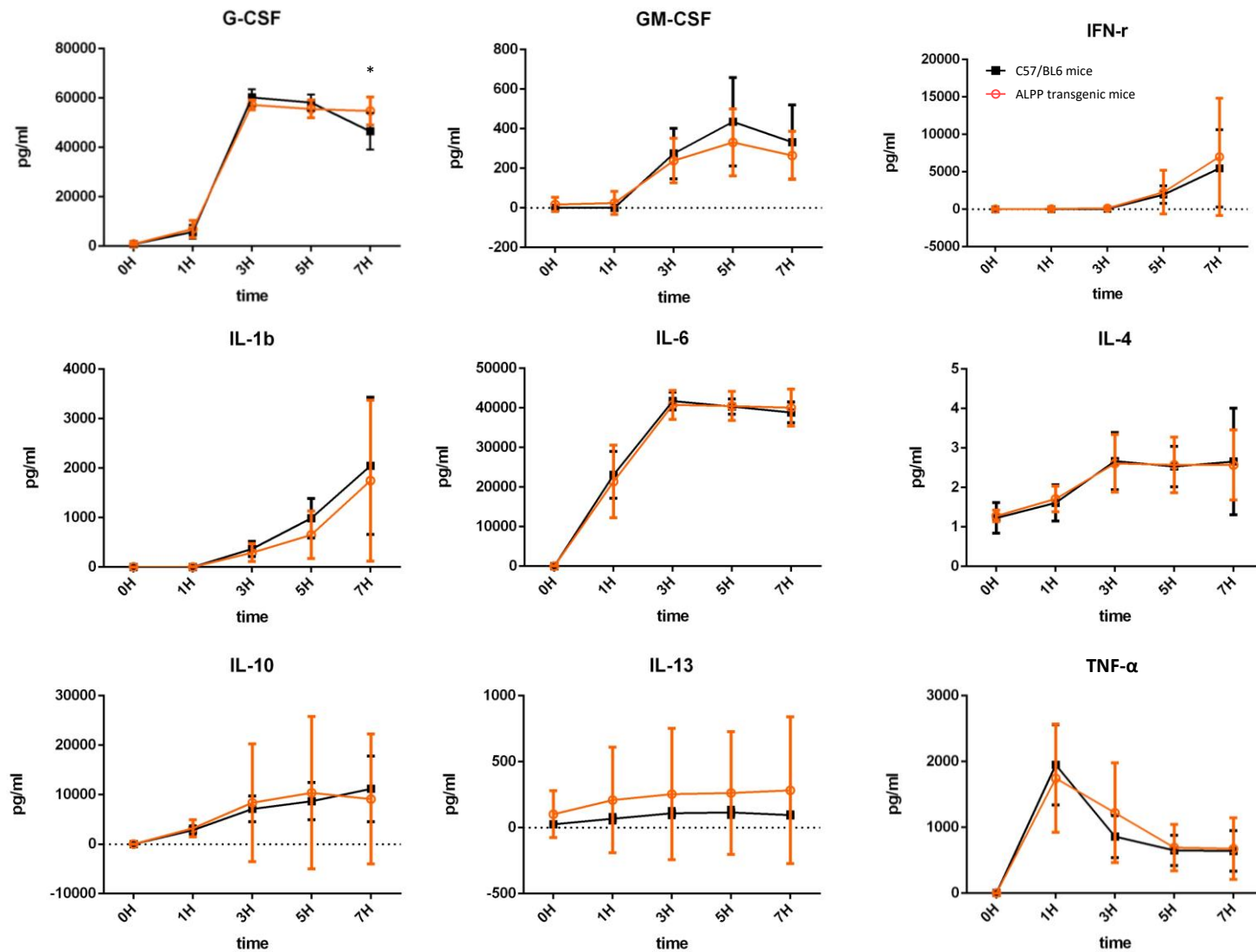

**Supplementary Fig. 3**

Supplement: Supplementary file 1 [file DataSheet1.pdf]
