# Supplementary material for: Evaluation of the immunological functions of placental alkaline phosphatase in vivo using ALPP transgenic mice
Source: Front Immunol. 2025 Feb 6;16:1499388. doi: 10.3389/fimmu.2025.1499388 (PMC11839614; doi:10.3389/fimmu.2025.1499388)
Supplement: Supplementary file 2 [file Table1.docx]

Supplementary Table 1 Summary of the reagent information used in the study

| Reagents | Company | Catalog No. | Lot No. |
| --- | --- | --- | --- |
| ALPP cDNA construct | OriGene Technologies, Inc | SC119167 |  |
| Recombinant ALPP protein | OriGene Technologies, Inc | TP310504 | 393273 |
| LPS | Sigma-Aldrich | L2630 | 099M4002V |
| Anti-ALPP Ab [ERP6141] | Abcam | ab133602 | GR323330-4 |
| pHrodo™ Green BioParticles® Phagocytosis Kit | Invitrogen | P35381 | 2462647 |
| THP-1 cell | ATCC | TIB-202™ |  |

Supplementary Table 2 Summary of the percentage of different immune cell subsets in wild-type and ALPP transgenic mice

| Immune cell subset | markers | | C57/BL6 mouse | ALPP transgenic mouse |  |
| --- | --- | --- | --- | --- | --- |
|  |  |  | % | % |  |
| T_reg_ cell | CD4^+^CD25^+^Foxp3^+^ | | 2.19 | 1.78 |  |
| CD4 | CD4^+^ | | 21.83 | 18. 72 |  |
| CD8 | CD8^+^ | | 11.07 | 12.74 |  |
| NK cell | CD335^+^ | | 1.29 | 1.47 |  |
| B lymphocytes | CD19^+^ | | 0.59 | 0.85 |  |
| Dendritic cells | CD4^+^CD11c^+^ | | 1.57 | 1.64 |  |
| Monocytes | CD4^+^CD11b^+^ | | 1.45 | 1.51 |  |
| Monocytes | F4/80^+^CD11b^+^ | | 2.43 | 2.38 |  |
| Stem cell | CD4^+^CD34^+^ | | 0.99 | 0.79 |  |
|  | | CD8^+^CD34^+^ | 1.04 | 0.85 |  |
